# Pediatric frequent relapsing nephrotic syndrome with multiple cerebral infarctions accompanied by patent foramen ovale and cerebral venous sinus thrombosis: a case report

**DOI:** 10.1186/s12882-024-03579-x

**Published:** 2024-04-24

**Authors:** Zentaro Kiuchi, Eriko Tanaka, Saaya Nunokawa, Sawako Yoshida, Akira Hosaki, Tomohito Kogure, Masami Narita

**Affiliations:** 1 Department of Pediatrics, Kyorin University School of Medicine, 6-20-2 Shinkawa, Mitaka-Shi, Tokyo, 181-8611 Japan; 2 Department of Cardiology, Tokyo Women’s Medical University, 8-1 Kawada-Cho, Shinjuku-Ku, Tokyo, 162-8666 Japan

**Keywords:** Nephrotic syndrome, Multiple cerebral infarctions, Cerebral venous sinus thrombosis, Patent foramen ovale

## Abstract

**Background:**

Idiopathic nephrotic syndrome (NS) presents as a hypercoagulable state, of which thromboembolism (TE) is a well-known life-threatening complication. Although TE is more likely to occur in venous vessels than arterial vessels, arterial TE is important because it may cause after-effects, including tissue necrosis and cerebral infarction (CI); therefore, prompt diagnosis and appropriate treatment are required. We report a pediatric NS case with multiple CIs.

**Case presentation:**

A 14-year-7-month-old Japanese girl was diagnosed with frequent relapsing NS, accompanied by headache and disturbance of consciousness during the second relapse. Brain magnetic resonance imaging (MRI) and four-dimensional computed tomography revealed multiple CIs, vasogenic edema, and cerebral venous sinus thrombosis (CVST). The patient had no underlying thrombophilia other than hypercoagulability due to NS and prednisolone (PSL), and no cardiac arrhythmia; however, a right-to-left shunt through the patent foramen ovale (PFO) was observed with the Valsalva maneuver by echocardiography. Therefore, we assumed that a potential cause of multiple CIs might be an embolic stroke, caused by thrombosis formed from a hypercoagulable state due to NS and PSL treatment and reached through PFO. Antiplatelet and anticoagulant therapies were administered for TE. She was treated with PSL and mycophenolate mofetil (MMF) for NS. Rituximab (RTX) was administered to prevent NS relapse after complete remission (CR). She underwent transcatheter PFO closure at age 14 years and 9 months because we considered that the right-to-left shunt through the PFO would be one of the risks for recurrent cerebral embolism when NS relapses. One year after the onset of CIs, an MRI indicated that the CVST had resolved, leaving no neurological sequelae due to CI; therefore, anticoagulant therapy was discontinued. And then she has been in CR for NS with only MMF therapy.

**Conclusions:**

CI is a serious complication in patients with NS. The pathogenesis of multiple CIs is various, including right-to-left shunt through PFO, in addition to the hypercoagulability due to NS. It is important to investigate and manage underlying risks such as PFO, besides preventing the relapses of NS by aggressive treatments using MMF and RTX, in patients with NS.

## Background

Complications of idiopathic nephrotic syndrome (NS) include infection, acute kidney injury, and thromboembolism (TE); among them, TE is a known life-threatening condition [1]. Venous TEs, such as deep vein thrombosis (DVT), pulmonary embolism (PE), renal vein thrombosis, and cerebral venous sinus thrombosis (CVST), occur in 3% of pediatric patients with NS. The underlying pathophysiology of TE in NS is multifaceted, including factors such as urinary leakage of important coagulation regulatory proteins, namely plasminogen, antithrombin III (AT-III), protein C, and protein S, and compensatory synthesis of proteins, namely macroglobulins, fibrinogen, thromboplastin, and factors II, V, VII, VIII, and X in the liver [2]. Glucocorticoids, which are key drugs for NS treatment, induce a hypercoagulable state that may cause TE. Although TE is more likely to occur in venous vessels than arterial vessels, arterial TE is important because it may cause after-effects, including tissue necrosis and cerebral infarction (CI) [3, 4]; therefore, prompt diagnosis and appropriate treatment are necessary. Herein, we report a pediatric NS case complicated with multiple CIs.

## Case presentation

A Japanese girl experienced edema in her lower extremities and weight gain at 14 years and 3 months of age. She had severe proteinuria, hypoalbuminemia, and hyperlipidemia. The patient was diagnosed with idiopathic NS and treated with oral prednisolone (PSL) (initial therapy, 60 mg/day for 4 weeks and subsequently, 40 mg every other day for 4 weeks). Dipyridamole was administered for the prevention of thrombotic complications. She successfully achieved complete remission (CR) on the 14th day after the initial onset.

**Fig. 1:**
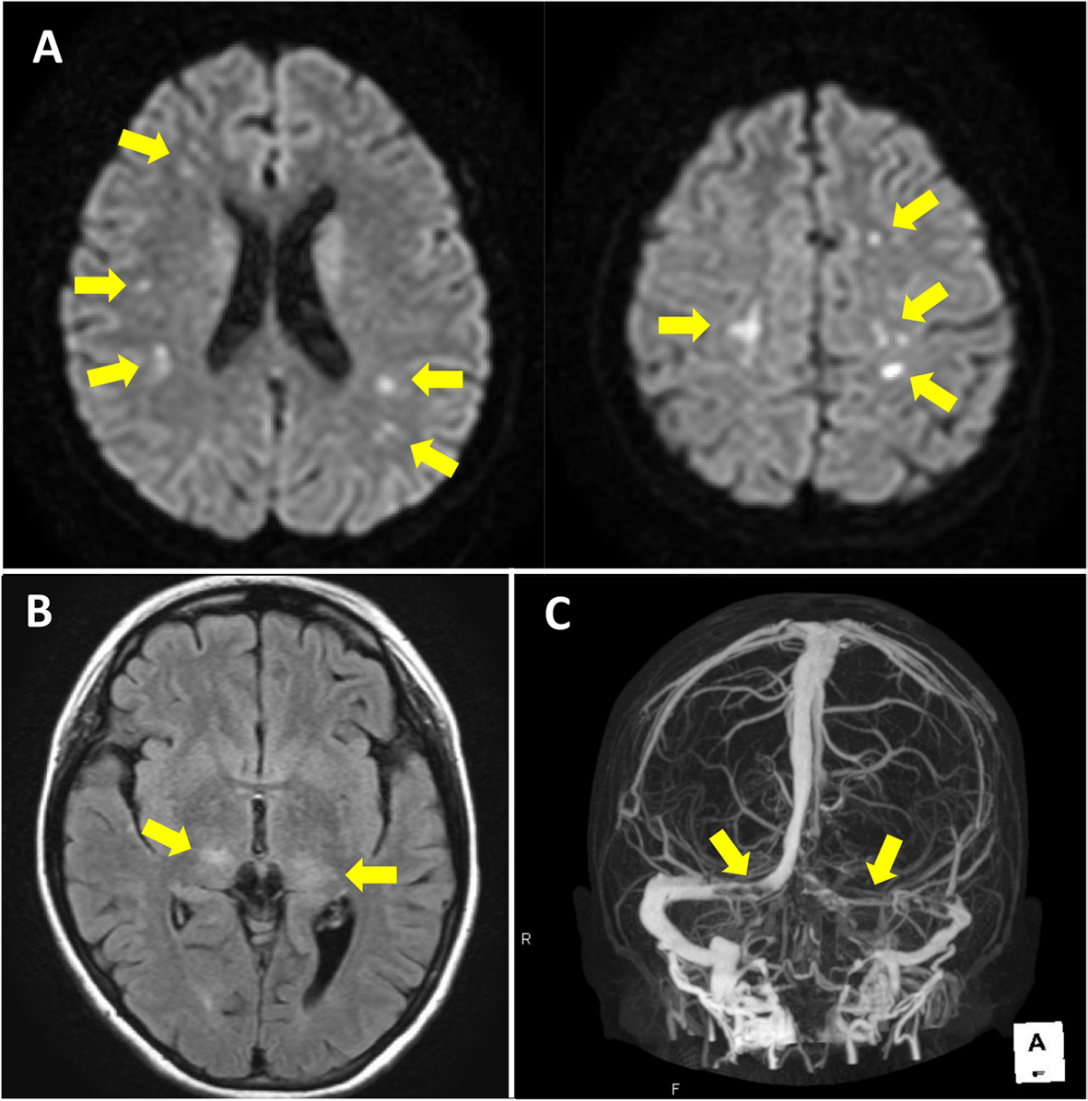
Magnetic resonance imaging (MRI) and computed tomography (CT) findings. **A** Diffusion-weighted MRI images showed acute phase multiple cerebral infarctions (arrows) mainly in the bilateral anterior cerebral artery/middle cerebral artery and middle cerebral artery/posterior cerebral artery watershed areas. **B** Fluid-attenuated inversion recovery MRI images showed faint hyperintensities in the bilateral thalamus (arrows), suggesting vasogenic edema. **C** Four-dimensional CT images showed occlusions due to thrombi in the rectus sinus, Galen vena cava, basal vein, and bilateral transverse sinus (arrows)

However, two NS relapses occurred 3 and 4 months later; therefore she was diagnosed with frequent relapsing NS. PSL was resumed at 60 mg/day, and dipyridamole was administered at 200 mg/day as well for both relapses because of the high risk of thrombosis due to a hypercoagulable state caused by NS and long-term PSL administration. On the 11th day after the second relapse, the patient gained 5 kg from her normal body weight of 42 kg due to edema and experienced abdominal pain and headache; subsequently, the patient was admitted to our hospital. Her laboratory examinations showed that the albumin level was 1.5 g/dL, the AT-III level was 58.8%, the D-dimer level was 9.3 μg/mL, and the spot urine protein to creatinine ratio was 12.8 g/g•Cr (selectivity index (SI): 0.18). Her blood pressure was maintained as 98/60 mmHg, however, there was intravascular dehydration as shown in the low inferior vena cava and aorta diameters ratio of 0.46 on abdominal ultrasound examination [5]. Supportive medications such as albumin and furosemide were administered to treat the edema and intravascular dehydration. Some kind of TE was suspected because of the increased D-dimer. Therefore, the AT-III agent was replenished to suppress hypercoagulable state. However, disturbance of consciousness (Glasgow Coma Scale; E3V4M5) appeared on the 13th day after the second relapse; therefore, magnetic resonance imaging (MRI) of the brain was performed because central nervous system disorders were considered. Multiple CIs were observed in bilateral watershed areas (Fig. 1A) on diffusion-weighted images. Fluid-attenuated inversion recovery images showed faint hyperintensities in the bilateral thalami, suggesting vasogenic edema (Fig. 1B). Additionally, four-dimensional computed tomography (CT) showed occlusions due to thrombi in the rectus sinus, Galen vena cava, basal vein, and bilateral transverse sinus, indicating CVST (Fig. 1C). The observed multiple CIs were microinfarctions and did not cause apparent neurological damage. The disturbance of consciousness was considered to result from reversible thalamic vasogenic edema due to CVST, especially deep cerebral venous thrombosis, including thrombosis of the straight sinus, vein of Galen, and basal vein [6, 7]. In addition to dipyridamole, anticoagulant therapy with intravenous heparin up to 14,000 units/day was initiated and activated partial thromboplastin time was maintained at 40–50 s. After two days, the state of consciousness improved, and no obvious paralysis was observed. Subsequently, she was switched to oral anticoagulant warfarin, and a prothrombin time-international normalized ratio of 2.0–3.0 was maintained.

**Fig. 2:**
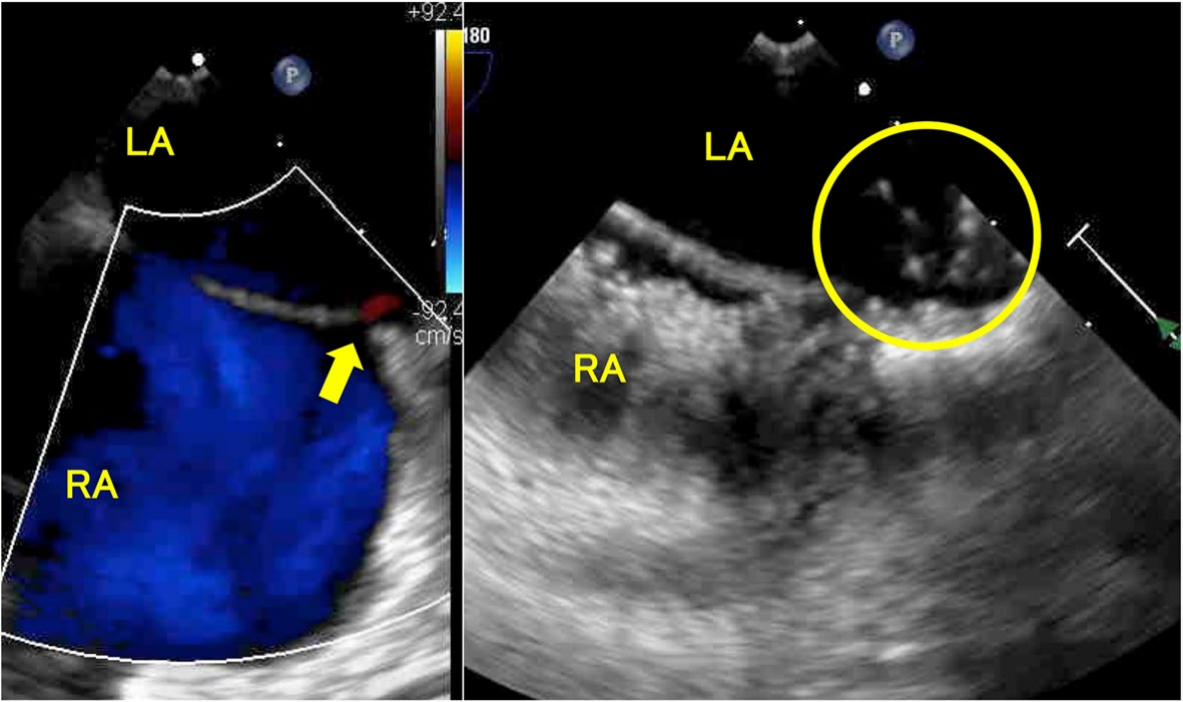
Transesophageal echocardiography findings. The left figure showed a 1.5 mm slit-like defect, patent foramen ovale in the fossa ovalis, and right-to-left shunt blood flow (arrow) observed during the Valsalva maneuver. The right figure showed the microbubbles (circle) from the right to the left atrium during the Valsalva maneuver. RA, right atrium; LA, left atrium

To search the causes of multiple CIs, we investigated other thrombosis, heart diseases, and underlying thrombophilia, which would be a risk factor for embolism, other than hypercoagulability due to NS and PSL. As a result, DVT, PE, carotid artery stenosis, vasculitis, and other thrombophilia were not detected (Table 1). Regarding heart diseases, no cardiac arrhythmias were observed on Holter electrocardiography; however, a patent foramen ovale (PFO) was suspected on transthoracic echocardiography. A 1.5 mm slit-like defect in the fossa ovalis and right-to-left shunt blood flow was observed during the Valsalva maneuver by using contrast transesophageal echocardiography (Fig. 2). There was no atrial septal aneurysm which was an independent risk factor for stroke.

**Table 1:** Examination results for thrombophilia investigation

	Results	Normal range	Units
**Protein C activity**	**125**	**70–140**	**%**
**Protein S antigen**	**64.1**	**60–150**	**%**
**Antinuclear antibody test**	** < 1:40**	** < 1:40**	**ratio**
**Anti-dsDNA antibody**	** < 0.6**	** < 10**	**IU/mL**
**Anti-Sm antibody**	** < 0.8**	** < 7**	**U/mL**
**Anti-SS-A (Ro) antibody**	**1.2**	** < 7**	**U/mL**
**Anti-SS-B (La) antibody**	** < 0.4**	** < 7**	**U/mL**
**Anticardiolipin IgG antibody**	** < 4**	** < 12.3**	**U/mL**
**Anti-beta 2 glycoprotein I antibody**	** < 1.3**	** < 3.5**	**U/mL**
**Lupus anticoagulant test**	**1.1**	** < 1.2**	

There was no decrease in protein C or protein S, which are blood coagulation regulators, and no increase in specific antibodies, which are characteristic of systemic lupus erythematosus, Sjögren’s syndrome, or antiphospholipid antibody syndrome. No underlying thrombophilia other than hypercoagulability due to NS and PSL was detected

For the treatment of frequent relapsing NS, mycophenolate mofetil (MMF) was initiated on the 14th day after the second relapse and increased to 1500 mg/day after five days. The patient achieved CR on the 25th day after the second relapse and PSL was reduced to 60 mg every other day after four days. And then rituximab (RTX) was administered at the age of 14 years and 8 months. However, the third relapse occurred on the 20th day after the PSL reduction. Due to the risk of TE, she was hospitalized and treated with intravenous heparin and oral dipyridamole therapy to prevent thrombi formation, whereas PSL was increased to 60 mg/day in addition to MMF. After achieving CR on the 8th day after the third relapse, the oral anticoagulant was switched to warfarin. The patient underwent transcatheter PFO closure using Amplatzer PFO occluder 25 mm (Abbott medical) at 14 years and 9 months of age because we considered that the right-to-left shunt through the PFO would be a risk for recurrent cerebral embolism when NS relapses and PSL treatment would be resumed. Oral aspirin was additionally initiated after PFO closure. Dipyridamole was discontinued thereafter. In this case, the bubble study by echocardiography at 14 years and 10 months of age showed complete PFO occlusion. And then aspirin was also discontinued. At 15 years of age, she was evaluated by MRI, the CVST was observed to have resolved, leaving no neurological sequelae due to CI. The second RTX was administered at 15 years and 1 month of age. Subsequently, oral warfarin anticoagulant therapy was successfully terminated at 15 years and 6 months of age. And then she has been in CR for NS with only MMF therapy.

## Discussion and conclusions

TE is well-known as a life-threatening complication of idiopathic NS. Our patient presented headache and impaired consciousness, and TE was suspected based on elevated thrombotic markers such as D-dimer. It has been reported that thrombosis may occur even in asymptomatic patients in NS. For example, PE was reported to be asymptomatically detected by ventilation perfusion scintigraphy in 27% of children in NS remission [8]. Zhang et al. revealed that subclinical PE occurred in 28% of pediatric NS patients without respiratory symptoms by dual-energy CT pulmonary angiography [9]. Therefore, NS may have venous thrombosis such as PE, even if they are asymptomatic; thus careful evaluation of several TE factors, including thrombotic markers such as D-dimer, is required. To our knowledge, there are no reports having investigated whether patients with NS are asymptomatic for DVT or CVST. The International Pediatric Nephrology Association guidelines mention that there is insufficient evidence to recommend routine prophylactic antithrombotic therapy during the nephrotic state in children and do not clarify the criteria for antithrombotic therapy for NS if preventive antithrombotic therapy is needed [10]. In our case, although dipyridamole was administered during the nephrotic state, TE occurred. The TE event in our patient was considered to result from hypercoagulation due to intravascular dehydration and hypoalbuminemia caused by NS relapse and long-term PSL treatment with high doses due to the timing of the first and second relapses being close. Thus, because NS patients have a high risk for TE events, it is expected that consensus standards will be established in the future for appropriate prevention and treatment of TE in NS patients.

We considered that important factors in the treatment of this patient were reducing the risk of thrombus formation by NS relapse and PSL use. It is necessary to select appropriate immunosuppressants to prevent NS relapse. We did not perform a renal biopsy for a histological diagnosis, because TE occurred during the second relapse, and antithrombotic therapy was initiated. However, our patient had a good response to PSL at the first onset and all relapses, with her SI of proteinuria showing highly selective, suggesting a minimal change nephrotic syndrome as a histological diagnosis. As standard treatments for frequent relapsing NS of minimal change disease, cyclosporine, MMF, and RTX are broadly used. Some studies reported that cyclosporine caused vascular endothelial cell damage and promoted thrombi generation [11]; thus, we considered that cyclosporine is not appropriate for a patient who experienced thrombosis. MMF and RTX treatment is highly effective for frequent relapsing NS that can reduce the frequency of relapse [12]. Therefore, we selected MMF and RTX in this case, and successfully achieved to prevent NS relapse.

Our patient was complicated by multiple CIs, thus other important managements were to consider the underlying pathophysiology of multiple CIs and to prevent recurrence of CI in the future. Generally, the main causes of multiple cerebral infarctions include obvious cardiogenic embolisms such as atrial fibrillation, atherothrombotic brain infarction, and lacunar infarction due to thrombotic occlusion of cerebral arterioles. However, none of them were related to our case. Hypercoagulable state due to NS and PSL use cannot be sufficient to cause multiple CIs. Thus, our case was classified as embolic stroke of undetermined source (ESUS) and we searched other underlying risks and found PFO. The ESUS is a subgroup of nonlacunar cryptogenic ischemic strokes in whom embolism was the likely stroke mechanism. Potential causes of ESUS generally include such as venous thrombosis with right-to-left shunt considering paradoxical cerebral embolism (PCE), arteriogenic emboli, minor-risk potential cardioembolic sources like valvular heart diseases, covert paroxysmal atrial fibrillation, and occult malignancy [13]. Therefore, we assumed the possibility of PFO being related to multiple CIs and decided to perform transcatheter PFO closure. We searched venous embolisms besides CVST. The patient was already under antithrombotic therapy at the time of investigation, which may have affected the result. Regarding PE investigations, only contrast-enhanced CT was carried out instead of pulmonary scintigraphy and it may lack the accuracy of examinations. Although the investigation had been performed in these conditions, we detected no embolisms other than CVST and concluded that the patient had no other potential risks of venous embolisms. There are rare reports of brain-to-brain embolism, where cerebral embolism is caused by intracerebral venous thrombosis through the right-to-left shunt [14, 15]. In this case, brain-to-brain embolism caused by CVST through PFO was also suggested as one of the causes of multiple CIs.

There are indications for the treatment of PFO-causing cryptogenic CI. PCE with PFO has previously been treated with antiplatelet drugs; however, in 2017, catheter therapy for PFO was reported to be more effective [16–18]. However, these studies were conducted on adults. Cerebral embolism through a PFO is very rare in pediatric patients; therefore, there are only a few cases in which catheter treatment has been indicated for PFO. Wawrzyńczyk et al. reported that seven children aged 12–16 years underwent successful transcatheter PFO closure, and no procedure-related complications were observed [19]. Although it is necessary to consider appropriate age, body size, risks, and period of postoperative antithrombotic therapy prior to transcatheter PFO closure in children, PFO should be actively managed by transcatheter closure in specific adolescents and young adults. In this case, the patient had a high risk of TE, which is one of the major reasons for choosing transcatheter PFO closure.

CI is a serious complication in patients with NS. The conceivable pathogenesis of multiple CIs is various, including right-to-left shunt through PFO, in addition to the hypercoagulability due to NS and PSL. In conclusion, we suggest that it is important to investigate and manage underlying risks of CI, such as PFO, besides preventing the relapses of NS by aggressive treatments using MMF and RTX, in patients with NS.

## Data Availability

All data generated during this study are included in this article.

## Abbreviations

AT-III: Antithrombin III
CI: Cerebral infarction
CR: Complete remission
CT: Computed tomography
CVST: Cerebral venous sinus thrombosis
DVT: Deep vein thrombosis
ESUS: Embolic stroke of undetermined source
MMF: Mycophenolate mofetil
MRI: Magnetic resonance imaging
NS: Nephrotic syndrome
PCE: Paradoxical cerebral embolism
PE: Pulmonary embolism
PFO: Patent foramen ovale
PSL: Prednisolone
RTX: Rituximab
SI: Selectivity index
TE: Thromboembolism
